# The Attractions of Agreement: Why Person Is Different

**DOI:** 10.3389/fpsyg.2019.00978

**Published:** 2019-05-22

**Authors:** Marcel den Dikken

**Affiliations:** ^1^Research Institute for Linguistics, Hungarian Academy of Sciences, Budapest, Hungary; ^2^Department of English Linguistics, Eötvös Loránd University, Budapest, Hungary

**Keywords:** agreement, person, number, agreement attraction, long-distance agreement, relativization, specificational copular sentences, concord

## Abstract

This paper establishes the generalization that whenever agreement with the finite verb is controlled by a constituent that is not in a Spec–Head relation with the inflectional head of the clause, this agreement cannot affect *person*. A syntactic representation for person inside the noun phrase and on the clausal spine is proposed which, in conjunction with the workings of agreement and concord, accommodates this empirical generalization and derives Baker’s Structural Condition on Person Agreement. The proposal also provides an explanation for the φ-feature agreement facts of specificational copular sentences. The paper places its findings on person vs. number agreement in the context of recent psycho- and neuro-linguistic investigation of number/person dissociation.

## Introduction

Agreement remains a highly complex matter, empirically as well as theoretically. With particular reference to agreement in specificational copular sentences, various ‘agreement attraction’, and long-distance agreement constructions, this paper addresses the question of why agreement phenomena systematically make a distinction between person and the other φ-features. Baker’s, (2008, 2011) Structural Condition on Person Agreement (SCOPA) was formulated to account for this, but by itself it offers no explanation for it. After a survey of the empirical territory I devote the core of the paper to deriving SCOPA and its effects from the syntactic representation of person in the noun phrase (as a specifier of the number phrase) and on the clausal spine (as a functional head in the complement of the number head), and from the workings of agreement and concord.

I close the paper by placing the findings regarding the difference in behavior between number and person agreement in the context of the recent psycho- and neuro-linguistic literature on number/person dissociation. Significant differences in behavior have been found between number agreement and person agreement in a suite of psycho- and neuro-linguistic studies — especially those conducted by Mancini and her co-workers on various Romance languages (see Mancini et al., 2011 for ERP experiments, Mancini et al., 2014b for self-paced reading experiments, and Mancini et al., 2017 for an event-related fMRI experiment). In their 2011 study on the ERP patterns evinced by subject-verb agreement violations in Spanish, number and person were found to differ in two ways: *(a)* person agreement violations give rise to N400 effects, which are ‘seldom reported in the literature’ (p. 69) for ‘mere’ agreement mismatches; and *(b)* person but not number agreement violations produce an early increased P600 effect at frontal (rather than posterior) sites. Mancini and colleagues interpret the frontal P600 effect as a reflex of ‘discourse-related integration difficulties’ (p. 73), and reinforce the semantic-pragmatic role played by person agreement by their understanding of the N400 effect (usually associated with problems of interpretation) as an indication that person mismatch causes an interruption of ‘the establishment of interpretive relations among constituents’ (p. 72) — particularly, of the association of the morphosyntactic person marking with the representation of the discourse participant (speaker/hearer) in the left periphery of the clause.

Mancini et al. (2017) recast their findings in terms of the postulation of two different mechanisms involved in agreement phenomena, which they call ‘feature-checking’ and ‘feature-mapping.’ Number and person agreement are argued to involve a common φ-feature-checking mechanism but to differ in their feature-mapping options, with number mapping to cardinality and person to the discourse. The present paper bears marginally on feature-mapping (the interpretive side of number and person marking), in its discussion of number agreement between the relativized head and the finite verb of a relative clause. But the main impact of this paper lies in what it has to say regarding Mancini and colleagues conclusion that number and person agreement share the same feature-checking mechanism. The material reviewed in this paper argues for a key difference between the feature-checking processes involved in number and person agreement: number agreement is possible under both Agree and the Spec–Head relation; person agreement, on the other hand, cannot transpire under (downward) Agree, being establishable only in a Spec–Head configuration.

## Person is Different

### Agreement in Specificational Copular Sentences

#### Specificational Pseudoclefts

It is often said that copula agreement distinguishes neatly and reliably between the predicational and specificational readings of pseudoclefts of the type in (1): Declerck (1988, p. 79), the source of these particular examples, asserts that (1a) is unambiguously specificational, and (1b) is predicational.^1^

(1)
  (a) what you have bought *is* fake jewels
  (b) what you have bought *are* fake jewels

But while it is true that (1a) only supports a specificational reading (equivalent to *you have bought fake jewels*), (1b) is not quite as unambiguous as Declerck makes it out to be. Similarly, in *what John brought was*/^?^ *were the crackers*, plural inflection on the copula is (marginally) possible on a specificational reading of the pseudocleft. Declerck (1988, pp. 79–80) himself points out that ‘[i]n specificational sentences the number of the copula can apparently be determined by that of either the superficial subject NP or the variable NP.’ The examples in (2a,b) are from Declerck, with his judgments (or those of his informants) provided; the ones in (2c–e) I have taken from Heycock (2012), with her original judgments included (see also Den Dikken, 2017).

(2)
  (a) what I need {*is*/^??^*are*} more books
  (b) what we can’t have here {*is*/^?^*are*} theft and robbery
  (c) what he saw behind him {*was*/*were*} two men
  (d) what makes something a pencil *are* superficial characteristics such as a certain form and function
  (e) all I could see {*was*/*were*} two staring eyes

No such oscillation is found for person, however: the sentences in (3) (also due to Heycock, 2012) are ungrammatical with person agreement between the copula and the post-copular focus.

(3)
  (a) what he saw behind him {*was*/*were} you
  (b) what makes this party go {*is*/*are} you
  (c) all I could see {*was*/*were} you

This is our first indication that number and person should be treated distinctly in the morphosyntax of English.

#### Double-NP Specificational Copular Sentences

In English double-NP specificational copular sentences such as (4)–(5), the copula agrees with the precopular noun phrase for both number and person (Heycock, 1992; Moro, 1997):^2^

(4) the biggest problem {*is*/*are} the agreement facts
(5) the biggest problem {*is*/*are} you

Dutch, German and Italian seem to return judgments that are the exact opposite of the ones reported for English: in (6)–(7), the copula must agree with the post-copular focus in both number and person.

(6)
  (a) de oorzaak van het ongeluk {*waren*/*was} kapotte remmen (Dutch)
  the cause of the accident were/was broken brakes
  (b) die Unfallsursache {*waren*/*war} defekte Bremsen
    (German)
  the accident-cause were/was defective brakes
  (c) la causa della rivolta {*sono*/*è} le foto del muro (Italian)
  the cause of.the riot are/is the pictures of.the wall
(7)
  (a) de schuldige {*ben*/*is} ik de schuldige {*ben*/*is} jij
    (Dutch)
  the culprit am/is I the culprit are/is you
  (b) der Schuldige {*bin*/*ist} ich der Schuldige {*bist*/*ist} du
    (German)
  the culprit am/is I the culprit are/is you
  (c) il colpevole {*sono*/*è} io il colpevole {*sei*/*è} tu
    (Italian)
  the culprit am/is I the culprit are/is you

For Italian, these facts are systematic. Moro (1997), who first discussed them in detail, has a syntax for them that makes them fall out without causing trouble for any extant account of agreement: *la causa della rivolta* in (6c) and *il colpevole* in (7c) are base-generated as left-adjuncts to IP, with a pro-predicate (*pro*) raising to the structural subject position; this *pro* copies the φ-features of the referential noun phrase of which it is predicated (i.e., the focus), so with I agreeing with *pro* we automatically derive full φ-agreement with the post-copular focus (8) makes this clear.

(8) [_IP_ *il colpevole* [_IP_ *pro*_i_ [_T_ copula_i_ [subject_i_ (…)]]]]

The Dutch and German facts are more problematic — first because (as Den Dikken, 1998 shows) they are not amenable to an account along Moro’s (1997) lines; and secondly because they are not nearly as straightforward as the Italian facts are. As a matter of fact, the examples in (6a,b) and (7a,b) are a red herring. For these root sentences, there are derivations available that treat the sentence-initial noun phrase as a topic in the left periphery and place the post-copular subject in the structural subject position, SpecIP. On such a derivation, the φ-agreement facts in (6a,b) and (7a,b) are parallel to the φ-agreement found in (9), Verb Second constructions with a non-subject in the left periphery and the subject occupying the structural subject position and agreeing with the finite verb.

(9)
  (a) op de vensterbank {*staan*/*staat} twee vazen (Dutch)
    on the window-sill stand.pl/stand.3sg two vases
  (b) bananen {*zul*/*zullen} je daar niet vinden
    bananas will.2sg/will.pl you there not find

To avoid the confounding effect of Verb Second, we should look at non-root clauses (which do not show Verb Second), as in Dutch (10) and (11):

(10)
  ze denken/betwijfelen dat de oorzaak van het ongeluk kapotte remmen {*waren*/*was}
  they think/doubt that the cause of the accident broken brakes were/was
(11)
  (a) ze denken/betwijfelen dat de schuldige ik {*ben/*is}
  they think/doubt that the culprit I am/is
  (b) ze denken/betwijfelen dat de schuldige jij {*bent/*is}
  they think/doubt that the culprit you are/is

The result is grammatical with number agreement but bad with person agreement. In the case of (11) this yields ineffability: with this linear order, I find that there is no φ-feature inflection on the copula that comes out grammatical.^3^ To get a grammatical output, we must refrain from predicate inversion, as in (11′), which has person agreement between the subject pronoun and the finite verb.

(11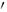)
  (a) ze denken/betwijfelen dat ik de schuldige {*ben*/*is}
    they think/doubt that I the culprit am/is
  (b) ze denken/betwijfelen dat jij de schuldige {*bent*/*is}
    they think/doubt that you the culprit are/is

These facts present us with two questions: *(i)* why is *person* agreement with the focus impossible when predicate inversion takes place, and *(ii)* why is agreement with the inverted predicate barred? Question *(i)* bears directly on the main theme of this paper, and will be answered the section entitled “Why Person Is Different.” The second question is strictly speaking tangential to my concerns here — but for completeness’ sake, I will address it briefly in the remainder of this section.

Heycock (2012):fn. (3) suggests that the oscillation between singular and plural number inflection on the copula seen in (2), repeated below, is ‘likely… due to the possibility of *what* and *all* (or the empty noun it modifies) being underspecified for number,’ which she thinks allows them to pick up their number specification from their associate (presumably under concord).

(2)
  (a) what I need {*is*/^??^*are*} more books
  (b) what we can’t have here {*is*/^?^*are*} theft and robbery
  (c) what he saw behind him {*was*/*were*} two men
  (d) what makes something a pencil *are* superficial characteristics such as a certain form and function
  (e) all I could see {*was*/*were*} two staring eyes

Heycock’s parenthesis ‘or the empty noun it modifies’ points us toward an answer to the question of why agreement with the fronted predicate in (10)–(11) is impossible. In Den Dikken (2006), it is proposed that in copular inversion constructions, what raises to the structural subject position is consistently a projection of a silent noun. Thus, double-NP copular inversion sentences such as those in (10) and (11) have a syntax of the following sort:

(12)
  [_IP_ [_PRED_ ∅ [*the cause*/*culprit*]]_i_ [_I_ I+relator = *be* [_RP_ focus [_R_ *t*_REL_ *t*_i_]]]]

With copular inversion constructions analyzed as in (12), the fact that the copula cannot φ-agree with the fronted predicate in (10)–(11) can be attributed to the absence of inherent φ-features on the silent noun. The fact that in double-NP specificational copular sentences such as my favorite authors are/*is Austen and Heller (from Heycock, 2009) we find plural inflection on the copula follows from the silent noun’s ability to show number concord (with either the conjoined subject of predication or plural *authors*): *the PERSONS who are my favorite authors are Austen and Heller*.^4^

### Special Agreement and Person

Before moving on to the analysis the section entitled “Why Person Is Different,” let me present a further set of contexts in which person agreement behaves markedly differently from number agreement: contexts that I will group together under the rubric of ‘special agreement.’

#### Agreement Attraction

The variants of the sentences in (13) and (14) with a plural-inflected finite verb (*think*, *are*) are well-known from the syntax and psycholinguistics literature (see Kimball and Aissen, 1971 on the former, and Bock and Miller, 1991 on the latter) as examples of number agreement between the finite verb of the clause and the ‘wrong’ target: in each case, finite verb agreement fails to target the entire subject of the clause; instead, agreement is ‘attracted’ to the relativized noun in (13) or to a subpart of the complex subject noun phrase in (14) (modeled on examples given in Kayne, 1998) — whence the name ‘agreement attraction’ [coined for cases of the type in (14), but apt for (13) as well].^5^

(13)
  (a) the people who Clark {*thinks*/^!^*think*} are in the garden
  (b) how many people {*does*/^!^*do*} Clark think are in the garden?
(14)
  (a) the identity of these people {*is*/^!^*are*} to remain a secret
  (b) these people’s identity {*is*/^!^*are*} to remain a secret

Like agreement in specificational copular sentences, agreement of this type involves *number*, not *person*: person agreement between the finite verb and a non-subject is impossible in English:^6^

(15)
  (a) I, who Clark {*is*/*am} hoping will marry his daughter
  (b) you, who Clark {*is*/*are} hoping will marry his daughter
(16)
  (a) the identity of me {*is*/*am} to remain a secret
    my identity {*is*/*am} to remain a secret
  (b) the identity of you {*is*/*are} to remain a secret
    your identity {*is*/*are} to remain a secret

#### Long-Distance Agreement

Baker (2011, sect. 2.3.3) points out that the kind of long-distance (cross-clausal) agreement found in Tsez (Polinsky and Potsdam, 2001; see fn. 1, above) likewise sets person apart — this time not just from number but from gender as well. Baker brings up the case of Loka̧a̧. In (17a), agreement between the matrix predicate and the object of the gerund that serves as its subject involves noun class (gender) and number; (17b) shows that such long-distance agreement is impossible for person.

(17)
  (a) [ȩ-sau kẹ-dẹi] e-tum ȩ-tawa (Loka̧a̧)
    7 -fish ger/5-buy 7 sg-be.very 7 sg-be.difficult
    ‘buying fish is very difficult’
  (b) *[min ke-funna] n-tum n-tawa
    1sg ger/5-surprise 1sg -be.very 1sg -be.difficult
    ‘surprising me is very difficult’

The facts in (13)–(17) solidify the conclusion reached in “Agreement in Specificational Copular Sentences” on the basis of the data of specificational copular sentences, and confirm the existence of an important dichotomy within the set of φ-features, setting person aside from the rest. The next section seeks to explain this dichotomy.

## Why Person is Different

### Structural Condition on Person Agreement

Baker (2008, 2011) codifies the specialness of person agreement as his SCOPA, reproduced in (18). Baker (2011, p. 877, fn. 3) suggests (building on but modifying Franck et al.’s, 2006 work on agreement) that ‘agreement for first- and second-person can never take place under mere Agree,’ but requires the Spec–Head relation. I believe this is on the right track. In “The Place of Person in the Structure of the Noun Phrase and on the Clausal Spine,” I will present an analysis of the place of person in the structure of the complex noun phrase and on the clausal spine which is mobilized in “The Syntax of Agreement: Agree Versus the Spec–Head Relation,” “Person Agreement as Attraction,” “Agreement Attraction “Long-Distance Agreement,” “Long-Distance Agreement,” “Copular Inversion and Agreement,” and “Relativization and Agreement” to explain how person agreement is different from number agreement, and to derive the main effects of SCOPA.

(18)
  Structural Condition on Person Agreement (SCOPA)
    a category F can bear the features +1 or +2 if and only if a projection of F merges with a phrase that has that feature and F is taken as the label of the resulting phrase.

### The Place of Person in the Structure of the Noun Phrase and on the Clausal Spine

For the functional heads for person (Harley and Ritter’s, 2002 class node participant) and number (Harley and Ritter’s individuation), I will henceforth use the Greek letter π and the symbol #, resp. Like Harley and Ritter, I will take π to exclusively make the distinction between speaker ([+author]) and addressee ([–author]). For ‘third person.’ the feature [–participant] can be assigned to the D-head of the nominal phrase. But importantly, ‘third person’ is not a possible specification for π.^7^ The following subsections address the place of π and # in the complex noun phrase (see “The Place of Person in the Internal Structure of the Noun Phrase”) and on the clausal spine (see “The Place of Person on the Clausal Spine”).

#### The Place of Person in the Internal Structure of the Noun Phrase

As a starting point, I will build up the structure of the complex noun phrase, along the lines of (19), which is effectively a ‘syntactic translation’ of Harley and Ritter’s (2002) feature geometry for the set of φ-features — person, number, and gender.^8^


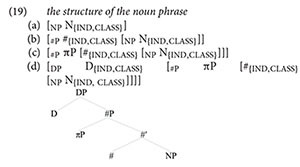


At the bottom of the noun phrase, we find a projection of the head noun, N. The gender specification of the noun (class, again following Harley and Ritter’s, 2002 terminology) is inherent to N. The noun is also specified for number, but its number properties are environmental, not genetic: the value for the feature [ind] is determined by a functional head labeled #, projecting outside NP. On top of #P, a projection for the definite determiner (D) can be built. This D-head establishes an Agree relation with # for [ind] and [class], which is how articles get specified for number and gender. Importantly, person is represented inside the structure of the noun phrase not as a head on the nominal spine but as a *specifier* in the nominal extended projection — the specifier of #P, to be precise. It occupies the same structural position (*mutatis mutandis*) as the subject of a clause: with D corresponding to C, and # corresponding to I, the πP in (19d) occupies the equivalent of SpecIP in the clause.

One thing that the proposal in (19) helps explain is the well-known fact (see Postal, 1966) that (20a,b) are grammatical while (20c) is not (regardless of the case form of the pronoun):

(20)
  (a) we/us linguists
    [_#P_ πP = *we/us* [#_{IND:PL, CLASS}_ [_NP_ N_{IND:PL, CLASS}_ = *linguists*]]]
  (b) you linguists
    [_#P_ πP = *you* [#_{IND:PL, CLASS}_ [_NP_ N_{IND:PL, CLASS}_ = *linguists*]]]
  (c) *they/them linguists

The pronouns in (20a,b) are interpreted as the subjects of the predicate *linguists*, with # as the relator of the predication relation (in the sense of Den Dikken, 2006). To be able to form a grammatical pronoun–noun construction of this sort, # must be present in the structure and explicitly specified for number to serve as a relator. In simple binary number systems such as English, ‘singular’ is absence of an explicit specification for number (i.e., a ‘bare’ class node [ind]). This explains the fact that (20a,b) do not have singular counterparts (*I linguist, *you linguist). And to be eligible for occupying Spec#P in (19), the pronoun must be specified for person, and no larger than πP.^9^ The English third person plural pronouns *they* and *them* fail to meet these requirements. I have taken the position that ‘third person’ never instantiates a feature specification for the person head π (which is only specifiable for [± author]), but instead is marked on D as [–part] (or not marked at all; see fn. 7). The fact that the English third person plural pronouns *they* and *them* are introduced by the same voiced dental fricative that represents the definite article (*the*) confirms that these pronouns project full-fledged DPs, too large for Spec#P. This explains why in English, *they* and *them* cannot be combined with the projection of a common noun, as in (20c).^10^

Cross-linguistically as well, first- and second-person pronouns show a tendency to be relatively small in size, whereas third-person pronouns pattern with DPs.^11^ Thus, in the Romance languages, while the third-person object clitic pronouns typically feature a token of the definite article (D = *l-*) in their morphology (cf. French *le* ‘him,’ *la* ‘her,’ *les* ‘them’), the first- and second-person clitics do not. And in Hungarian (21), where full DPs and third-person pronouns serving as objects invariably trigger definiteness inflection on the transitive verb, first- and second-person object pronouns combine with indefinite inflection, due to their limited size (no larger than #P).

(21)
  (a) szereted a fiút / őt (Hungarian)
    love.2sg.def the boy.acc (s)he.acc
    ‘you_SG_ love the boy/him/her’
  (b) szeretsz minket
    love.2sg.indef us.acc
    ‘you_SG_ love us’
  (c) szeretünk titeket
    love.1pl.indef you_PL_.acc
    ‘we love you_PL_’

In the structure of nominal expressions, person/[part] finds itself in the specifier position of number/[ind]. Inside the noun phrase, there is agreement for number and gender, but never for [part]. Similarly, # (spelled out by the indefinite article, simple numerals, perhaps certain existential quantifiers) inflects for number and gender, but never for [part]. That D, # and N share their specifications for number and gender is a straightforward reflex of the fact that all three are in an Agree-chain (‘head-head agreement’; cf. also ‘feature inheritance’ or ‘extended projection’), all having matching number and gender properties. The πP, as a left branch, is not a member of this chain.

#### The Place of Person on the Clausal Spine

On the clausal spine, # and π are also separate entities. But this time around, they find themselves in a complementation configuration, with the #-head embedding πP as its complement:^12^

(22)
  [_CP_ C [_#P_ #_{IND}_ [_P_ π_{PART}_ (…) [_VP_ V_{IND, PART}_]]]]

In the clause, the finite verb shows agreement with the subject for number and person. The fact that person is a player in the clausal agreement system (unlike inside the noun phrase) indicates that it must be able to serve as a probe, adorned with unvalued feature [*u*part]. This motivates the decision to represent π as a head on the clausal spine. Number has that status as well, bearing [*u*ind]. In addition, the head # is responsible for the assignment of nominative case to the subject. Nominative case is associated with φ rather than tense (as we know from inflected infinitives with nominative subjects in Portuguese; Raposo, 1987). For reasons discussed in the section entitled “The Syntax of Agreement: Agree Versus the Spec–Head Relation,” the clausal π-head cannot serve as a probe in (downward) Agree relations, so in constructions in which the nominative subject appears below the inflectional domain (sentences with ‘VP-internal subjects’) it is inevitable to pin the nominative case feature on #. In constructions in which the nominative subject appears in the structural subject position (‘SpecIP’), it surfaces in the higher of the two φ-related functional projections in (22): the contrast between *probably he isn’t the culprit* and *probably isn’t he the culprit shows that, with *is* in the higher inflectional head, the nominative subject *he* must be placed to its left, in Spec#P. This in turn tells us that # is structurally higher than π (something that, for Indo-European, is impossible to verify on morphological grounds: person and number form portmanteaux in the verbal inflectional system of IE). The #-over-π structure in (22) is further supported on the basis of the syntax of number and person agreement in these languages, as I will now show.

### The Syntax of Agreement: Agree Versus the Spec–Head Relation

The hypothesis that person is represented as a specifier in the noun phrase and as a functional head on the clausal spine below number has important consequences for the distribution of person agreement in the clause.

In the structure of the noun phrase, person is not represented on D or #.^13^ So how does person agreement in the clause come about? Let us first examine (downward) Agree. The person head on the clausal spine has nothing to probe for: the πP of the pronominal subject in the verbal core is not directly accessible to the clausal π-head because it is contained within the pronominal subject, occupying the specifier position of the subject, which is itself a specifier. Subparts of specifiers are not directly accessible to higher probes: specifiers are merged into the structure as fully built structural chunks (see Uriagereka, 1999 for the origins of this idea); no outside probe can by itself reach into the innards of a specifier. So the clausal π-head cannot directly target the πP inside the subject. The clausal π-head cannot target the entire subject pronoun (i.e., #P) integrally either because #P, specified for [ind] but not for [part], is not a match for the π-head’s [*u*part] feature. So person agreement cannot happen under (downward) Agree.^14^ And since the clausal π-head cannot probe the pronominal subject of the clause, it cannot attract it to its specifier position either, so it also cannot establish a Spec–Head relation with the pronominal subject in the clausal πP.

But the next higher head, #, does manage to Agree with and attract the pronominal #P, provided that the clausal π-head with its [*u*part] feature gets out of the way. Locality of probing makes it impossible for the #-head’s [*u*ind] feature to probe past an intervening unvalued feature [*u*part] on the clausal spine.^15^ But if the clausal π-head raises and adjoins to #, then # will find a match without obstruction: it can engage in an Agree relation with the pronominal #P; and if the EPP so dictates, the clausal #-head can also attract the pronominal #P to its specifier, which results in a Spec–Head relation between the clausal #-head and the pronominal subject. This Spec–Head relation involves not just number but person as well. The clausal π-head must raise to # in order for # to be able to attract the pronominal subject to Spec#P, adjoining to # and forming a complex probe [_#_ π [#]] with it. Under the Spec–Head relation between this complex probe and the subject, a total match between the two must be forged, in concert with (23) (from Den Dikken and Dékány, 2019; see also Guasti and Rizzi, 2002; Shlonsky, 2004, p. 1496; Franck et al., 2006 for relevant facts and discussion):

(23)
  the total match constraint on Spec–Head agreement
    feature checking under the Spec–Head relationship requires total matching of the features of the head and the features of its specifier.

Under (downward) Agree, the functional head # probes the subject just for its own unvalued [*u*ind] feature. But once the #-head has probed the subject and attracted it to the specifier of the complex probe [_#_ π [#]], a total match must be established between this probe and the subject, by (23). The probe–goal relation between the clausal #-head and #P lifts the opacity of the latter, rendering the πP in the specifier position of the pronominal subject an accessible goal to the π-portion of the complex probe.^16^ The structure in (24) illustrates, for first- and second-person pronominal subjects.

(24)
  [_#P_ [_#P_ πP_{PART: AUTHOR}_ [_#
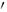
_ #_{IND}_ [_NP_ N]]]_i_ [_#
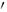
_ [_#_ π_{uPART}_ [#_{uIND}_]]… *t*… *t*_i_…]]

The result of (24) is agreement for both number and person, with the latter contingent on the former, as desired: it is impossible for the finite verb of a clause to agree with a pronoun in person but not in number, but the converse is possible. Directly relevant to the unidirectional contingency relation between person and number agreement are the facts in (25) (Akmajian, 1970, p. 154), involving highest-subject relativization, and (26) (Baker, 2011, p. 887), illustrating Kimball and Aissen (1971)-type relatives in which the head (a non-subject within the relative clause) attempts to control agreement with the finite verb of the relative clause.

(25)
  (a) I, who am tall, was forced to squeeze into that VW
  (b) we, who are/*am tall, were forced to squeeze into that VW
(26)
  (a) *I, who Clark am hoping will come,..
  (b) ^!^we, who Clark are hoping will come,..

The ungrammaticality of (25b) with *am* tells us that person agreement in the absence of number agreement is illegal. And the fact that *are* is possible (for speakers who have ‘Kimball and Aissen effects’) in (26b) indicates that the verb can agree with the head in number without agreeing in person: after all, from the ungrammaticality of (26a) [recall (15a)] we learn that person agreement between the finite verb of the relative clause and the head of the relative is impossible when the head is not the finite verb’s subject.

To summarize, there can be no person agreement under downward Agree between the clausal π-head and the πP of the subject pronoun because, the latter being encapsulated inside an opaque #P, π cannot itself peek inside the subject and target its specifier (πP). But, provided that π raises to #, the clausal #-head can probe the entire subject pronoun, #P, and attract it to its specifier. Once #P has been probed, its specifier becomes accessible, and hence, in compliance with the constraint in (23), which demands that all the features of the complex probe [_#_ π [#]] find a match, the π-portion of this complex probe values the [*u*part] feature of the subject’s πP-specifier. The raised π-head must probe the subject when the latter is in Spec#P. By contrast, when the subject does not raise, π cannot probe it when π is *in situ* (because #P is not a match for it, and the subject’s πP is not accessible); and when π moves and adjoins to #, it lies dormant as an inactive subpart of [_#_ π [#]] unless it is activated by the constraint in (23), which applies only when [_#_ π [#]] is in a Spec–Head relation with the raised subject. From this it emerges that person agreement with pronominal subjects is possible if and only if the subject is in a Spec–Head relationship established in the #P on the clausal spine. Person agreement under (downward) Agree is impossible.

### Person Agreement as Attraction

In the approach taken in the section entitled “The Syntax of Agreement: Agree Versus the Spec–Head Relation,” person agreement between the finite verb and a first- or second-person pronominal subject involves a relationship of feature valuation targeting the specifier of the structural subject, itself occupying a specifier position [see (24), repeated below]. This reminds us of agreement attraction cases of the type in (27b) [recall (14b), above]. Like (24), (27b) instantiates an agreement relation between the finite verb and the specifier (here, the possessor) of the structural subject: see (28).

(27)
  (a) these people’s identity is to remain a secret
  (b) ^!^these people’s identity are to remain a secret
(28)
  [_#P_ [_DP1_ DP2_{–PART, IND:PL}_ [_D
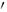
_ D_{–PART, IND}_… [_NP_ N]]]_i_ [_#
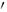
_ [_#_ π_{uPART}_ [#_{uIND}_]]… *t*… *t*_i_…]]
(24)
  [_#P_ [_#P_ πP_{PART:AUTHOR}_ [_#
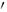
_ #_{IND}_ [_NP_ N]]]_i_ [_#
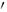
_ [_#_ π_{uPART}_ [#_{uIND}_]]… *t*… *t*_i_…]]

In (24), the clausal π-head gets a chance to agree with the πP embedded in the pronominal subject thanks to the fact that the #-head of the clause values its [*u*ind] feature against that of the #P in its specifier. Similarly, in (28) the #-head gets a chance to value its [*u*ind] feature against the [ind:pl] specification of the possessor DP2 embedded in the possessive DP2 thanks to the fact that the π-portion of the complex probe [_#_ π [#]] can establish a feature-valuing relationship with the [–part] feature on the head of the possessive DP1, opening it up for # probing the plural feature of DP2. With (27b) commonly referred to as a case of agreement attraction, we come to the conclusion that person agreement with first- or second-person subject pronouns is a form of agreement attraction.

This is a *prima facie* rather surprising conclusion in light of the fact that whereas (27b) is usually considered an error, person agreement with the subject is perfectly flawless. Why does person agreement not have the acceptability status of familiar agreement attraction cases? The answer lies in competition. In the case of person agreement (24), there is just a single [part]-specified node in the Spec–Head domain of the complex probe [_#_ π [#]], meeting no competition and serving as the only possible match for the probe’s [*u*part] feature. In (28), on the other hand, there are two instances of [ind] present in the complex subject: one on DP1 and another on DP2. Each is a potential match for an agreement relation with the finite verb. When such competition presents itself, the structurally closest agreement relation is the unmarked one. In (27a), the clausal #-head agrees directly with DP1 in the structure in (28); in (27b), valuation of [*u*ind] is postponed until after π-probing has opened up DP1 and made DP2 available as a goal for #. The unmarked option of these two is (27a); (27b) is the marked case. But in the case of (24), there is no competition — indeed, probing the πP in Spec#P is the clausal π-head’s only chance (its last resort, if you will) at getting its [*u*part] feature valued. Hence markedness does not come into play in (24).

### Agreement Attraction

The bulk of the literature on number agreement attraction effects has concentrated, not on cases in which the attractor occupies the ‘Saxon genitive’ position [as in (14b)], but instead on cases in which the attractor is contained in a post-nominal PP or relative clause, as in (14a) [adapted from Kayne, 2000, and repeated here as (29a)], (29b,c) (Bock and Miller, 1991) and (29d) (Dillon et al., 2013).

(29)
  (a) the identity of these people {is/^!^are} to remain a secret
  (b) the key to the cabinets {is/^!^are} rusty
  (c) the path to the monuments {is/^!^are} littered with bottles
  (d) the new executive who oversaw the middle managers {was/^!^were} dishonest about the company’s profits.

In Den Dikken (2001), I suggested (following Kayne, 1998) that the DP-contained plural makes its way up to SpecDP (the ‘Saxon genitive’ position) at LF, via an operation akin to or identical with Quantifier Raising. This would help account for the distributive interpretation of (29a) (for each person, there is a different identity) and possibly of (29b) as well. But a QR-style approach does not carry over to (29c,d), for which there is neither a Saxon-genitival paraphrase nor a distributive reading — and at any rate, QR out of a relative clause would be syntactically very difficult to uphold. I will not pursue this line of thinking further, therefore.

For (29a–c), the idea that probe–goal relations make otherwise opaque domains transparent (Den Dikken, 2018; recall fn. 16) may be put to good syntactic use, with the clause-level π-probe agreeing with the subject-DP for [–part] and allowing the #-probe to target the DP-contained plural noun phrase. But for (29d), it is inconceivable that the matrix #-head could be given syntactic access to the plural object of the relative clause construed with *executive*. For examples of this type, it seems to me vanishingly likely that syntax could assist in providing an account. So although syntax can make major strides in the understanding of agreement attraction, there remains to my mind an irreducible residue of linear string effects in the realm of agreement attraction phenomena. (Relevant here as well is the discussion of Dillon et al., 2017 at the end of section “Feature Sharing in Non-subject Relativization: The Kimball and Aissen Facts Revisited,” below.)

But neither structurally nor linearly is the person specification of a subpart of the complex subject ever local to the finite verb. As a consequence, agreement attraction never involves person, as we saw in (16) (repeated below): the πP embedded inside the specifier of the clausal #-head cannot be engaged in an agreement relation with the #-adjoined π-head of the clause.

(16)
  (a) the identity of me {is/*am} to remain a secret
    my identity {is/*am} to remain a secret
  (b) the identity of you {is/*are} to remain a secret
    your identity {is/*are} to remain a secret

In (30), I illustrate the structure of the second example in (16a). The clausal #-head’s [*u*ind] can find a match in the number specification for DP1, the possessive noun phrase. And π can value its [*u*part] feature against DP1’s [–part], contributed by the D-head. The result of these feature valuations is *my identity is to remain a secret*, which is grammatical. As π finds a match in [–part] (‘third person’) on DP1, it cannot probe beyond this point. Hence, the possessor’s π_[PART:+AUTH]_ never comes into the picture. Even though both (30) and (24) (the latter repeated once more below, for ease of direct comparison) feature a πP in the specifier domain of the clausal [_#_ π [_#_ #]] probe, only in (24) is this πP accessible to the π-portion of the complex probe: in (24), the specifier of the clausal #-head is not itself specified for [part], enabling π to pick the person specification of the subject pronoun as its goal; but in (30), DP1 bears [–part], rendering a valuation relationship between the clausal π-head and the person features of DP1’s pronominal possessor impossible.

(30)
  [_#P_ [_DP1_ [_#P_ πP_{PART:+AUTH}_ [_#
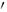
_ #_{IND}_…] [_D
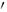
_ D1_{–PART, IND}_…]] [_#
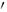
_ [_#_ π_{uPART}_ [#_{uIND}_]]…]]
(24)
  [_#P_ [_#P_ πP_{PART:AUTHOR}_ [_#_ #_{IND}_ [_NP_ N]]] [_#_ [_#
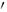
_ π_{uPART}_ [#_{uIND}_]]…]]

For the versions of (16) in which the personal pronoun occurs in a post-nominal *of*-phrase, agreement between the finite verb and the person feature of the pronoun is also impossible. Syntactically, the fact that the container-DP is specified as [–part] once again renders a probe–goal relation between the clausal π-head and the pronoun’s πP impossible. And because the pronoun’s πP is the specifier of the pronominal #P, it is not linearly adjacent to the finite verb either. All avenues toward person agreement attraction in constructions of the type in (16) are thus blocked, as desired.

### Long-Distance Agreement

Now that we have an answer to the question of why person-agreement attraction fails in (16), let us verify that long-distance person agreement of the type in (17b) [repeated below, along with grammatical (17a)] is also correctly ruled out.

(17)
  (a) [ȩ-sau kẹ-dẹi] e-tum ȩ-tawa (Loka̧a̧)
    7 -fish ger/5-buy 7 sg-be.very 7 sg-be.difficult
    ‘buying fish is very difficult’
  (b) *[min ke-funna] n-tum n-tawa
    1sg ger/5-surprise 1sg -be.very 1sg -be.difficult
    ‘surprising me is very difficult’

The number feature of *ê-sau* ‘fish’ in (17a) is directly represented on DP, and accessible to the complex [_#_ π [_#_ #]] probe in the matrix clause after the π-portion of this probe has established a feature valuation relation with CP, which I assume is, like D, specified for [–part].^17^ But the person feature of *min* in (17b) is not a possible goal for the matrix π-probe: after π has valued its [*u*part] feature against CP’s [–part], it is no longer active as a probe. The structures in (31a) and (31b) (in which I treat the gerund as the structural of the matrix clause^18^) illustrate, for (17a) and (17b), respectively.

(31)
  (a) [_#P_ [_CP_ [_DP_ D_{–PART, IND}_…]
    [C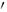 C_{–PART}_…]] [_#_ [_#_ π_{uPART}_ [#_{uIND}_]]…]]
  (b) [_#P_ [_CP_ [_#P_ πP_{PART: AUTHOR}_ [_#_ #_{IND}_ [_NP_ N]]]
    [_C
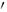
_ C_{–PART}_…]] [_#
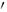
_ [_#_ π_{uPART}_ [#_{uIND}_]]…]]

### Copular Inversion and Agreement

Next, let us take a closer look at the specificational copular sentences of the section entitled “Agreement in Specificational Copular Sentences.” In these sentences, person agreement with the post-copular subject of predication is impossible. The examples in (32) and (33) (repeated from above) show this clearly.

(32)
  (a) all I could see {*was*/*were*} two staring eyes
  (b) all I could see {*was*/*were} you
(33)
  (a) *ze betwijfelen dat de schuldige ik ben (Dutch)
    they doubt that the culprit I am
  (b) *ze betwijfelen dat de schuldige jij bent
    they doubt that the culprit you are
  (c) ze betwijfelen dat de schuldige Jan is
    they doubt that the culprit Jan is

This again falls out from the proposal in “The Syntax of Agreement: Agree Versus the Spec–Head Relation,” given the analysis of inverse specificational copular sentences first presented in Moro (1997) and developed in further detail in Den Dikken (2006), according to which their syntax involves fronting of the underlying predicate into the structural subject position, as illustrated in (34):

(34)
  (a) [_SC=RP_ [subject] [_R
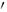
_ relator [predicate]]]
    ⇒ predicate inversion ⇒
  (b) [_TP_ [predicate]_i_ [_T
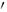
_ T+relator = *be*
    [_SC=RP_ [subject] [_R
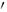
_ *t*_REL_ *t*_i_]]]]

Predicate inversion results in a syntactic structure in which the only way in which the copula can establish an agreement relationship with the post-copular subject is via (downward) Agree. Agree with the entire post-copular subject, as in (32a) and (33c), is perfectly fine; but person agreement with a subpart of the post-copular subject (in particular, with its πP) is impossible, for reasons discussed in “The Syntax of Agreement: Agree Versus the Spec–Head Relation.”

It also follows from the approach to syntactic agreement taken in this paper that in contexts of the type in (35b) and (36b), agreement attraction is impossible even for number. In the a-sentences, the noun phrase of *people* is the specifier of the specifier of the clausal #-head, just as in (27b), whose structure was given in (28). But in (35b) and (36b), the noun phrase of *people* is not in an agreement relation with #. This plural noun phrase is invisible to # both under the Spec–Head relation and for Agree purposes: though it is in the c-command domain of the probe #, the #-head can Agree directly only with the complex singular possessive noun phrase as a whole, which leads unequivocally to singular verb inflection.

(35)
  (a) two people’s silhouette {*was*/^!^*were*} all I could decipher
  (b) all I could decipher {*was*/*were} two people’s silhouette
(36)
  (a) these people’s information {*is*/^!^*are*} the cause of the computer glitch
  (b) the cause of the computer glitch {*is*/*are} these people’s information

The prediction made by the proposal accords well with the facts: while *are* is possible in the a-examples under attraction, it does not work at all in the copular inversion constructions in (35b) and (36b).^19^

### Relativization and Agreement

Finally, I will now return to the Kimball and Aissen (1971) facts, further enhanced by Baker (2011). The key contrast here is between (37a,b) and (37c) [adapted from (13) and (15), above]:^20^

(37)
  (a) I, who Clark {is/*am} hoping will be the finalist,..
  (b) you, who Clark {is/*are} hoping will be the finalist,..
  (c) these people, who Clark {is/^!^are} hoping will be the finalists,..

While the finite verb in the relative clause can be attracted to the number specification of the head of the relative clause, its person feature cannot be matched by the finite verb when the head is not its subject.

This observation is significant because, as we saw already in (25) [repeated below as (38)], when the head is the subject of the relative clause, person agreement between the head and the finite verb of the relative clause is grammatical:

(38)
  (a) I, who am tall, was forced to squeeze into that VW
  (b) we, who are/*am tall, were forced to squeeze into that VW

But even when the head is itself a subject, its person agreement behavior has an interesting twist: as Morgan (1972, p. 284) points out, long-distance relativization makes person agreement with the verb in the downstairs clause impossible:

(39)
  *I, who John says (the FBI thinks) am an anarchist/responsible,…

The empirical picture for person agreement under relativization is further complicated when we take the case form of the head of the relative clause (determined in the external syntactic context) into account. Kimball and Aissen (1971, p. 241) note that number agreement between the non-subject head and the finite verb is possible even when the head is not in a nominative case environment in the matrix clause:

(40)
  Mark knows/wants to talk to the people who Clark {thinks/^!^think} are in the garden

For number agreement between the head and the finite clause in cases of highest-subject relativization, the case-form of the head is also inconsequential, as (41) shows. However, Akmajian (1970) notes that person agreement in this context is possible only if the head is itself nominative: see (42).

(41)
  he had the nerve to say that to them, who have made him what he is today
(42)
  *he had the nerve to say that to me, who have made him what he is today

What I would like to present in this section is a comprehensive account of this entire picture. To my knowledge, this has never been undertaken previously. Analyses of the facts in (38) and (39) are themselves quite few and far between (since Akmajian, 1970; Ross, 1970; Morgan, 1972 first unearthed them, the generative literature has largely set them aside, with a moderate resurgence of attention in recent works by Heck and Cuartero, 2012; Douglas, 2015). But as far as I am aware, these subject relativization data have never been coupled with an analysis of the (extended) Kimball and Aissen facts.^21^

The following are the key players in the discussion to follow:

*(a)* the representation of person and number in the complex noun phrase presented in “The Syntax of Agreement: Agree Versus the Spec–Head Relation”
*(b)* the properties of the relative operator *who*
*(c)* an analysis of relativization involving predication inside the noun phrase (Den Dikken, 2006)
*(d)* feature sharing between the relative CP and the head noun phrase under concord
*(e)* feature sharing between CP, its head C, and the inflectional system of the clausal spine

With these players, we can gain a complete understanding of the facts in (37)–(42). The account is entirely deterministic, based in its entirety on assumptions defended in the foregoing and standard or independently plausible ingredients of the theory.

#### The Featural Specification of *Who* as Relative Operator

Let me begin by stating and supporting my assumptions regarding the featural specification of the relative operator *who* (which converge with those in Douglas, 2015, *contra* Heck and Cuartero, 2012).

The operator *who* projects a DP. As we know from the section entitled “The Place of Person in the Structure of the Noun Phrase and on the Clausal Spine,” D is not specifiable for the features [part: ± author] (i.e., for first- or second-person): πP finds itself on a left-branch position inside the structure of the complex noun phrase, and its specification for [part] does not ‘percolate’ up to D. We expect it to be universally impossible for *wh*-operators to be inherently marked for [part: ± author]. I assume that inherently, the D-head of *who* is radically unspecified for [part].

But D is specifiable for the feature [ind]. In English *wh*-questions, *who* is systematically singular (who is/*are coming?, who is/*are eligible?) unless it is in a predication relation with a plural-marked nominal, as in *who are the finalists?* (a question enquiring about the identity of the individuals to which *the finalists* applies, not a question asking the interlocutor to name the property that the finalists share — the latter would require the use of *what* as the *wh*-operator). In light of the fact that *who*, even in English *wh*-questions, can be plural-marked under the appropriate circumstances, I assume that the *wh*-word *who* is capable of bearing the feature specification [ind:pl]. (I return in the section entitled “Feature Sharing in Non-subject Relativization: The Kimball and Aissen Facts Revisited” to the way in which this comes about.) In relative clauses with a plural-marked human head, it is this plural-specified *who* that serves as the relative operator.

#### The Syntax of Relativized Noun Phrases

My outlook on the syntactic structure of relativized noun phrases is anchored in Den Dikken’s (2006) general theory of the syntax of predication. In this theory, relations that are traditionally treated in terms of modification and its structural correlate of adjunction are brought into the predicational fold, with adjectival attributive modification constructions of the type in (43a) involving reverse predication (i.e., a structure in which the predicate finds itself in the specifier position of the relator phrase), and their counterparts in (43b) being instances of canonical predication (with the predicate in the complement of the relator head).

(43)
  (a) the visible stars [_RP_ [_AP_ A] [_R_ relator [_#P_ # [_NP_ N]]]]
    the responsible person
  (b) the stars visible [_RP_ [_#P_ # [_NP_ N]] [_R_ relator [_AP_ A]]]
    the person responsible

For relative clause constructions, this procures a straightforward analysis, with the relative clause in the position of AP in (43b), as shown in (44).

(44)
  the stars that are visible
    [_RP_ [_#P_ # [_NP_ N]] [_R
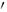
_ relator [_CP_ relclause]]]
    the person who is responsible

The difference between restrictive and non-restrictive relativization can be made in familiar terms, as a function of the size of the relativized constituent (i.e., the nominal in SpecRP). I will not take a specific stand on this issue. I will say only that the familiar ban on restrictive relativization of first- and second-person pronouns can be made to follow if restrictive relatives are necessarily in the scope of the D-head whereas non-restrictives are not: recall from “The Place of Person in the Internal Structure of the Noun Phrase” that first- and second-person pronouns are mere #Ps, hence ineligible for restrictive relativization except when a D is merged with them, as in *the me you’re seeing now is different from the me people see in public* (see also fn. 22).

#### The Raising Approach to Relativization Cannot Make the Right Cut

From (44) it is apparent that I am adopting a head-external approach to relative clauses: the head does not originate inside the relative clause. The person facts reviewed in the introduction to this section supply us with a cogent argument against existing head-internal or ‘raising’ analyses of relativization, at least for non-restrictive relatives with a first-person pronominal head.

Both Kayne’s (1994) version of the raising approach and Bianchi’s (1999) development thereof treat the head of the relative clause and the relative operator (*who* or *which*, depending on the humanness of the head) as a single constituent. At some point before the end of the syntactic derivation, the head moves around the relative operator into SpecDP, which is its terminus for Kayne; Bianchi subsequently splits the head and the relative operator apart via onward movement of the head into a position in the high left periphery of the relative clause. But such onward movement happens well and truly after the DP in (45) has already vacated its A-position in the clausal core — the structural subject position in the cases under consideration here. So the difference between Kayne’s and Bianchi’s versions of the raising analysis is of no consequence to us here: the two analyses share (45a) and (45b).

(45)
  (a) [_DP_ [_D
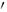
_ D = *who*/*which* head]]
  (b) [_DP_ head_i_ [_D
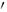
_ D = *who*/*which t*_i_]]

Let us investigate what the predictions made by (45) are for person agreement with the head.

To make the examination easier, (46) presents an update of (45) for the specific case of a first-person relativized head:

(46)
  (a) [_DP_ [_D
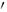
_ D = *who* [_#P_ πP_[PART:+AUTHOR]_ [_#
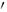
_ #…]]]]
  (b) [_DP_ [_#P_ πP_[PART:+AUTHOR]_ [_#
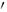
_ #…]]_i_ [_D
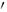
_ D = *who t*_i_]]

An immediate question we face is whether the movement of the head to SpecDP happens before or after the DP has made its way into the Â-domain of the relative clause. There is no immediately obvious answer to this question; so I will do the exercise of verifying the possibility of person agreement with the head for both logically possible scenarios. If the head remains *in situ* in the complement position of D while the DP is still in the A-domain of the clause, we get (47a); if movement to SpecDP happens early, we get (47b).

(47)
  (a) [_#P_ [_DP_ [_D_ D = *who* [_#P_ πP [_#_ #…]]]] [_#_ [_#_ π [_#_ #]]..
  (b) [_#P_ [_DP_ [_#P_ πP [_#
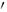
_ #…]]_i_ [_D
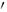
_ D = *who t*_i_]] [_#
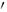
_ [_#_ π [_#_ #]]..

Person agreement between the complex probe [_#_ π [_#_ #]] and the pronoun contained inside the subject-DP will be possible in (47a) and (47b) provided that the πP of the pronoun, which is quite deeply embedded in the subject, can be made accessible to the π-portion of the complex probe. We can give the π-part of the probe access to πP inside the subject only if the complex probe establishes a feature-valuation relationship with DP and #P. The #-portion of the [_#_ π [_#_ #]] probe can value its [*u*ind] feature against that of DP, and D and # share their [ind] specification within the extended nominal projection (‘feature inheritance’ or ‘head-head agreement’). By the logic of Den Dikken’s (2018) theory of locality, this should probably be sufficient to render both DP and #P transparent for the purposes of a probe–goal relationship between the clausal π-probe and the πP inside DP. I have assumed (see “The Featural Specification of Who as Relative Operator”) that the D-head of *who* is itself radically unspecified for [part]. So provided that the #-portion of the clausal [_#_ π [_#_ #]] probe matches its [*u*ind] feature against that of D, it should be technically possible for the π-portion of this probe to match the [part:+author] specification of the πP inside DP in the structures in (47), regardless of whether movement of #P to SpecDP happens early or late in the derivation.

This sounds like good news for the analysis of the examples in (38), repeated below, where first-person agreement in conjunction with number agreement is obligatory inside the relative clause.

(38)
  (a) I, who am tall, was forced to squeeze into that VW
  (b) we, who are/*am tall, were forced to squeeze into that VW

But the problem for the raising analysis is that it makes person+number agreement with the head of the relative clause behave the same way in highest-subject relatives such as those in (38) and in long-distance relativization cases. As we know from (39), agreement with the head actually fails in examples of long relativization.

(39)
  *I, who John says (the FBI thinks) am an anarchist/responsible,…

The fact that long-distance relativization cannot give rise to person agreement on the finite verb of the clause of which the head is the subject is unexpected on the raising approach, assuming that (47) can deliver person agreement in principle. The head of the relative clause originates, on the raising approach, in the subject position of the most deeply embedded clause, where we know that, in the absence of relativization, it would certainly control person agreement with the finite verb; and we also know from (38) that in highest-subject relatives the complex structure in (47) that the raising analysis postulates within the relative clause manages to control full agreement with the finite verb.

I conclude, based on (39), that at least for non-restrictive relatives with a first-person head, a raising analysis is not tenable.^22^ I will work hereinafter with (44), taken from Den Dikken (2006).

#### Feature Sharing Between the Head and the Relative Clause: Concord

In the structure in (44), the relative clause and the projection of the head are in a predication relationship. Predication relations are well-known to give rise to feature sharing between the predicate and its subject. In the Russian example in (48a), for instance, the predicative adjective is inflected for the same gender, number, and case as its subject.^23^ This feature-sharing relationship between predicates and their subjects is commonly referred to as concord. Den Dikken and Dékány (2019) argue explicitly that concord does not involve the syntactic relationship of Spec–Head agreement. The short version of the argument is that concord (unlike Spec–Head agreement) frequently does not involve complete matching of features: there can be case concord without φ-concord; and there can be φ-concord without case concord. The examples in (48b,c) demonstrate this for Russian.

(48)
  (a) devočka krasivaja (Russian)
    girl.f.sg.nom beautiful.f.sg.nom
    ‘the girl is beautiful’
  (b) devočka byla krasivoj
    girl.f.sg.nom was beautiful.f.sg.inst
    ‘the girl was beautiful’
  (c) eti fakty problema
    these fact.m.pl.nom problem.f.sg.nom
    ‘these facts are a problem’

From this, I conclude that concord in predication structures (relator phrases) does not involve the syntactic relationship of agreement — it is not a feature-valuation relation but instead a copying operation, arguably [see esp. (50), below] taking place in the post-syntactic component (i.e., at PF).

More specifically, for cases of full concord such as (48a), I will assume that the sum total of the features present on the subject is copied over to the predicate. In the specific case of a relativized noun phrase, full concord takes place between the head and the relative CP in a structure of the type in (44). This causes CP to have exactly the same φ- and case-feature set as the head:


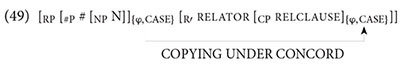


Concord targets the full set of features of the head of the relativized noun phrase as a batch, regardless of where these features are represented in the internal syntax of the nominal constituent — blind, that is, to the question of whether the person feature is represented on the syntactic head of the relativized nominal or only on its specifier (as in the case of first- and second-person pronouns). In the post-syntactic component, with internal syntactic bracketing erased, the head of the relative clause is one single cluster of features. All of the relativized head’s features will thus be involved in concord — including [part: ± author].

Since C is the head of CP, by standard feature percolation along X-bar projection lines this entire feature set is present on C as well. C and I (i.e., #+π) are in a feature-sharing relationship (‘feature inheritance,’ ‘head-head agreement’), so the local I has the head’s features communicated under concord as well. It is via this concordial chain that the local I of a highest-subject relative clause ends up agreeing with the head for all φ- and case-features.

This predicts that person agreement between the subject-head and the finite verb of the relative clause is possible only when the head has the appropriate case (i.e., nominative): otherwise there is a clash with I. As we saw in (42) [see again (50a)], this prediction is borne out, in a structural accusative case context. It is worth emphasizing that person agreement in the relative clause remains ungrammatical in environments in which the accusative case form of the head is not the fruit of a structural case-assignment relationship but instead the default case (see Schütze, 2001), as in (50b) (Akmajian, 1970) and (50c) (not previously discussed in the literature, to my knowledge).

(50)
  (a) he had the nerve to say that to me, who {has/*have} made him what he is today
  (b) it is me who {is/*am} responsible
  (c) (A) who’s going to climb up the ladder?
  (B) definitely not me, who {has/*have} vertigo
  (B′) me, who {has/*have} vertigo, climb up that ladder?! no way!

Neither in *it*-clefts (50b) nor in fragment answers (50c.B) or ‘*Mad Magazine* sentences’ (50c.B′) does the syntax assign structural accusative case to the pronoun. The accusative case form of the pronoun is not the exponent of a structural accusative case feature valued in the course of the syntactic derivation: the default accusative is a purely phonological (PF) property of the pronouns in question. Concord is a PF operation, so it copies not just structural accusatives but also default accusatives over onto the relative clause and, ultimately, onto the I-head of the relative clause, which has a nominative case feature. Resulting in a feature clash at I, the result of this copying is correctly rejected, not just in (50a) but also in (50b,c).

Unlike concord for person, number agreement between the head of a highest-subject relative clause and the finite verb is not ruled out in non-nominative environments [see (41)] because, as I mentioned in the section entitled “The Featural Specification of Who as Relative Operator,” the relative operator *who* is specifiable for [ind:pl] independently of concord. Person-feature sharing between the head and the finite verb, by contrast, is entirely dependent on concord, which entails case-feature identity.^24^

Though, as we have seen, the concord relationship between the head of the relativized noun phrase and the relative CP can stretch all the way down to the I-domain of the relative clause (via the feature-sharing relation between C and the local I), it cannot reach beyond this point. It is entirely impossible for concord to penetrate a clause embedded inside the relative clause: there is no path from the matrix I down into the subordinate clause along which the cluster of features of the head could be copied into the lower clause and reach its I-domain. In non-highest-subject relative clauses, in fact, the I of the relative clause itself is in a feature-valuation relationship with the subject of its clause, which is not the *wh*-operator linked to the head. So concord between the head of the relativized noun phrase and the I-domain is restricted to highest-subject relatives; the I of a clause embedded within the relative CP cannot be the beneficiary of a concord relationship between the head and the relative CP.^25^ This explains the locality effect seen in (38) vs. (39): I in the lower clause in (39) can only get default person inflection (‘third person’). Note that, because *who* is itself specifiable for [ind:pl], independently of concord, it is expected that number agreement should be possible in the downstairs clause — as is indeed the case: (51) is grammatical (see Douglas, 2015).

(51)
  we, who John says (the FBI thinks) are anarchists/responsible,..

#### Feature Sharing in Non-subject Relativization: The Kimball and Aissen Facts Revisited

With the results of the discussion in the previous section in mind, let us return to the facts noted by Kimball and Aissen (1971) and Baker (2011):^26^

(37)
  (a) I, who Clark {is/*am} hoping will be the finalist,..
  (b) you, who Clark {is/*are} hoping will be the finalist,..
  (c) these people, who Clark {is/^!^are} hoping will be the finalists,..

Concord does not help create person agreement in these kinds of sentences: the I-domain of the relative clause is in an Agree relationship with the subject of the relative clause (*Clark*) in syntax, which values I’s person feature — the DP of *Clark* is specified as [–part]. Concord between the head and the relative clause could not interfere with this. By the time that the effects of concord could kick in (at PF), the φ-features of the relative clause’s finite verb have already been fixed. The null hypothesis is that PF cannot undo or override specifications for φ-features established by valuation under Agree in syntax. So for (37a,b), concord would come too late: it cannot impose the head’s [part: ± author] specification onto the finite verb of the relative clause (already valued as [–part] by *Clark*) anymore.

Things are different in the case of number, for which the relative operator *who* is inherently specifiable. The feature [ind:pl] can be present on *who* in the syntax of the relative clause, and under the right circumstances, it can impose itself on the finite verb of the relative clause. There are, logically speaking, three points in the structure at which number agreement between the finite verb and *who* could come about in the case of (37c): in CP, under Spec–Head agreement; in IP, with *who* as an adjunct to or outer specifier of IP (in the case of densely successive-cyclic movement), again under a form of Spec–Head agreement; or under (downward) Agree, when the *wh*-operator is adjoined to the phase in the complement of I. In each of these configurations, the finite verb should be able to establish a feature-valuation relationship with *who*’s [ind:pl]. For my purposes in this paper, it does not matter which of these options is the right one. I will leave the matter open.^27^

One thing that I think is worth noting is that the ungrammaticality of *are* in (37b) not only confirms that person agreement is impossible in Kimball and Aissen-style constructions but also compels us to be precise about the number-matching relationship between the head of the relative clause and *who*, the relative operator. The null hypothesis for English *you*, whose form does not covary with the number of addressees, is that its morphological feature specification is constant regardless of its reference. Since *you*, when it is itself the subject of a finite verb, always triggers a plural form of the verb (also in the case of the copula: *you are*), this leads Kayne (2000) to assume that *you* is morphologically plural even in contexts of singular reference. Adopting this assumption leads to the conclusion that the number specification for the operator *who* in the relative clause is based on semantic numerosity, not morphological number. We know from (37c) that *who*, when specified as [ind:pl], is capable of controlling plural agreement in the relative clause. The fact, then, that plural agreement on the finite verb (*are*, the blanket plural form of the present-tense copula) is impossible in (37b) tells us that English *you*, when it has a singular referent [as is clear in (37b) from the form of the predicate nominal, *the finalist*], cannot be construed with [ind:pl]-specified *who* in the relative clause. The data in (52) (from Douglas, 2015, p. 36) make the same point for highest-subject relatives:

(52)
  (a) he had the nerve to say that to you_SG_, who {has/*have} made him what he is today
  (b) he had the nerve to say that to you_PL_, who {have/*has} made him what he is today.

In highest-subject relatives with a non-nominative pronominal head (where person agreement is impossible, for reasons discussed in “Feature Sharing Between the Head and the Relative Clause: Concord”), there is a clear difference between (52a) (with a single addressee) and (52b) (with a plurality of addressees) in the inflection on the finite verb. We know from (42) that person agreement with the pronominal head is excluded when the head is in a non-nominative environment (because concord between the head and the C–I cluster of the relative clause would result in a case clash in this context); so *have* in (52b) is a reflex of number agreement alone, between the finite verb and the relative operator *who*. The question is what determines the number specification of this *who*.

If, as Kayne (2000) argues, *you* is always morphologically plural, the fact that ‘singular *you*’ resists construal with plural *who* is surprising if the number specification for *who* is determined on the basis of morphological feature matching. After all, ‘singular *you*’ then has what it takes, morphologically, to license plural *who*. This does not necessarily mean, however, that Kayne’s morphological analysis of *you* is ill-founded (which is what Douglas, 2015, p. 36 takes the facts in (52) to show). What the ill-formedness of *are* in (37b) and the distribution of *has* and *have* in (52) show, on Kayne’s approach to *you*, is that the determination of the number specification for the relative operator *who* is based, not on the morphological number specification (i.e., the [ind]-feature) of the head of the relative clause, but on the numerosity of the referent of the head of the relative clause. Succinctly put, on Kayne’s analysis of English *you*, these inflection facts would have to be a reflex of ‘semantic agreement.’ The question of whether ‘semantic agreement’ exists and how it works is by no means an easy one to answer (see, e.g., Wechsler, 2011 and references cited there). I will not take a stand on the matter because it is orthogonal to my concerns in this paper. But the lie of the land is clear: for those who believe independently that ‘semantic agreement’ exists and is applicable in the context of relativization, the facts reviewed above do not pose a threat to Kayne’s (2000) argument that English *you* is always grammatically plural; but to those who reject ‘semantic agreement’ (in general, or in the specific context at hand), these facts suggest that English has two homophonous forms of the second-person pronoun *you*, only one of them morphologically specified as [ind:pl].

One final remark is in order. I have argued in this section that concord is not at play in Kimball and Aissen-style number agreement cases of the type in (37c), nor in examples such as (52b): the plural number inflection of the finite verb is determined in these cases by the [ind:pl] of *who* itself. This leads us to expect that plural agreement cases of these types should be replicable in *wh*-questions as well, with *wh*-operators that are specified as [ind:pl]. Consider the pair in (53)–(54):

(53)
  which people {are/*is} hoping Clark will be a finalist?
(54)
  which people {is/^!^are} Clark hoping will be finalists?

The *wh*-phrase *which people*${}_{\left[\mathrm{IND}:\mathrm{PL}\right]}$ of course controls plural agreement with the finite verb of the *wh*-question when serving as its subject, as in (53). But Kimball and Aissen (1971, p. 245) already showed that *wh*-questions can also give rise to agreement attraction, as shown in (54) (recall also (13)). Interestingly, however, Richard Kayne tells me that he rejects such agreement in questions with *who*, even when *who* is construed through predication with an explicitly plural nominal in the lower clause, as in *who* {does/*do} *Clark think will be the finalists*?. This should be investigated further: it is likely to be revealing regarding the precise circumstances (incl. possible locality restrictions) under which a [ind:pl] specification can be assigned to the bare *wh*-operator *who* under concord.

Dillon et al. (2017) report on a recent series of experiments they ran on Kimball and Aissen-effects in English *wh*-questions.^28^ Their results show that although ‘mismatch effects are largest when the *wh*-object is adjacent to the verb’ (p. 86), string adjacency between the finite verb and the *wh*-constituent is nonetheless ‘neither necessary nor sufficient to generate a mismatch effect’ (p. 85). The answer to the question of whether the structural subject or the *wh*-constituent controls finite verb agreement turns out to be determined for the most part by syntactic (configurational) factors. Dillon et al. (2017) review the spectrum of extant theoretical approaches to agreement attraction errors (in terms of feature transmission, subject confusion, and syntactic interference), and the results of their extensive empirical studies land them on the side of syntactic interference approaches such as Franck et al. (2006). This is a conclusion I welcome. However, Dillon and colleagues also caution that the role played by the Spec–Head relation may be less robust than Franck and colleagues (and the present paper) have made it out to be: in particular, Dillon et al. (2017, p. 80) ‘failed to find any reliable effect of preposition fronting on [their] ratings’ — agreement attraction was rated roughly equally in (55a) and (55b):

(55)
  (a) which trees {is/^!^are} the hiker resting under?
  (b) under which trees {is/^!^are} the hiker resting?

Future research should look into this at greater length, against the background of syntactic analyses of PP pied-piping (see esp. Heck, 2008 for important discussion of the syntax of pied-piping).

Beyond the P-stranding/PP pied-piping dichtomy, Dillon et al.’s (2017) study leaves room for additional experimentation regarding the nature and scope of Kimball and Aissen-style effects as well. I would particularly encourage future work that juxtaposes *wh*-relatives and *wh*-questions in a way that makes them more directly comparable. All of and colleagues five acceptability-judgment experiments involve root *wh*-questions, with subject–auxiliary inversion, which maneuvres the finite verb into a position in between the *wh*-constituent (in SpecCP) and the structural subject (in SpecIP). This makes these *wh*-questions different from relative clause constructions, which do not feature movement of the finite verb to C. A direct comparison of *wh*-relatives with non-root *wh*-questions, leveling the playing field with regard to the placement of the finite verb, would be able to tell us with more precision whether the ‘Kimball and Aissen effect’ is the same or different, qualitatively and/or quantitatively, in relatives and *wh*-questions.

### Summary

After this (unavoidably) rather elaborate discussion of agreement in relative clause constructions, let me summarize our findings in this section regarding person versus number agreement.

I started out by giving an explicit syntax for the representation of person inside the noun phrase and on the clausal spine. For first- and second-person pronouns, a structure was presented in which person (π) projects a phrase occupying the specifier position of number (#). This not only gives us a natural syntax for pronoun–noun constructions such as *us linguists*, but also paves the way for an explanation for the range of ways in which person behaves differently from number in the realm of agreement phenomena. Effectively, whenever person agreement is possible in syntax, it comes about in a configuration that is similar to the number agreement attraction effect seen in ^!^*these people’s identity are to remain a secret*. Both can materialize only when the subject is in a Spec–Head relation with the inflectional cluster on the clausal spine. This gives us an account of the majority of person agreement contexts, and explains the restrictiveness of person agreement in comparison to number agreement (without, however, giving person agreement the flavor of an attraction error).

For cases of person agreement between the pronominal head of a relativized noun phrase and the finite verb inside the relative clause, an appeal to a feature-sharing relationship different from Agree or Spec–Head agreement needed to be exploited: concord, a post-syntactic copying operation. The feature bundle of the head of the relativized noun phrase can be copied wholesale onto its predicate (the relative clause), and can make it from there to the inflectional cluster of the relative clause, but not beyond. Via this feature-copying process, full feature-sharing between the head and the finite verb becomes possible in highest-subject relatives — but not in long-distance subject relativization constructions, nor in cases of non-subject relativization. Number agreement between the finite verb of the relative clause and the head is always possible, giving rise to attraction effects in the case of long-distance and non-subject relativization, *à la* Kimball and Aissen (1971). That number never comes up empty-handed is thanks to the fact that the English relative pronoun *who* is specifiable for plural number (probably under ‘semantic agreement,’ as discussed in “Feature Sharing in Non-subject Relativization: The Kimball and Aissen Facts Revisited”). But as a DP, *who* is not specifiable for first- or second-person: [part: ± author] is exclusively the province of a πP in the specifier position of #P.

The main effects of SCOPA have now been successfully derived.^29^

## Conclusion

The empirical spotlight in this paper has been on ‘out of the ordinary’ agreement phenomena and the circumstances under which they are found in Universal Grammar. A key property common to all ‘agreement attraction’ and ‘long-distance agreement’ cases is that they cannot involve first- or second-person. It is this person restriction (hitherto poorly understood) that has been at center-stage here.

Baker’s, (2008, 2011) SCOPA captures the specialness of person, but because it is a condition that is itself left underived, it cannot explain it. I have derived SCOPA from *(a)* the syntactic representation of person in the noun phrase (with person structurally represented as a *specifier*) and on the clausal spine (with person and number each projecting X-bar structures, the former’s phrase embedded in the latter’s) and *(b)* the workings of agreement and concord. Central to the syntax of *(b)* is a distinction between (downward) Agree and Spec–Head agreement, with only the latter capable of effecting number and person agreement conjointly.

This, in combination with the representation of person in the internal structure of pronouns, successfully rules out all person agreement attraction and long-distance person agreement, as desired. The Spec–Head relation creates just the sort of niche needed for person agreement where it is legal.

With Franck et al. (2006), this paper thus affirms the existence of two syntactic mechanisms for the establishment of φ-feature agreement: Agree and the Spec–Head relation. Agree is more liberal in the sense that it can potentially establish long-distance agreement dependencies, whereas Spec–Head relations are by definition more local. But in another way, Agree is also more restrictive: it is impossible for an Agree-probe to target a subpart of a specifier in its c-command domain; so whenever we find agreement dependencies between a head and a subpart of a specifier (including cases of person agreement with subjects), a Spec–Head configuration must be involved.

Mancini et al. (2017) also argue for the postulation of two different mechanisms involved in agreement phenomena, which they call ‘feature-checking’ and ‘feature-mapping.’^30^ Number and person agreement are argued to involve a common φ-feature-checking mechanism (‘of which the similar left-anterior negative effect could be evidence’; p. 142) but to differ in their feature-mapping options, with number mapping to cardinality and person to the discourse (‘which would be behind the different posterior negative effect elicited by the two violations’; p. 142).

I have little to add to Mancini and her co-workers’ findings regarding the interpretive side of person marking (what they call ‘feature-mapping’), and find their interpretation thereof (which appeals to the representation of the speaker and the hearer in the left periphery) eminently plausible. For the discussion in the present paper, this is of no immediate concern. More to the point of the current discussion is Mancini et al.’s (2017) conclusion that number and person agreement share the same feature-checking mechanism. If what I have argued in the foregoing is on target, the feature-checking processes involved in number and person agreement are not, in fact, systematically identical: while number agreement is possible under both Agree and the Spec–Head relation, the facts reviewed above suggest that person agreement is established exclusively under the Spec–Head relation. The neurological measurements reported in Mancini and colleagues work do not suggest that there is a grammatical-processual difference between number and person agreement — but this may very well be an effect of the choice of constructions and languages studied: preverbal and silent subjects in Romance pro-drop languages (Italian, Spanish). It is likely that preverbal subjects and pro-dropped subjects in these languages are systematically in a Spec–Head relation with the T-head. Recall that both number and person agreement are possible under the Spec–Head relation. To probe into the question of whether the feature-checking mechanism(s) involved in person and number agreement are neurologically different, one would need to look at data like the ones studied in the present paper. These data define a research agenda that can take the interesting results of the work done by Mancini’s team further.

The picture resulting from the present paper (in particular, the division of labor between Agree, the Spec–Head relation, and the post-syntactic copying operation called concord) is principled and descriptively adequate in the complex realm of agreement attraction and long-distance agreement constructions discussed in this paper. The attractions of agreement are a boundless resource for morphosyntacticians. My hope is that the perspectives on the workings of agreement and the structural representation of the φ-features within the noun phrase will prove their mettle well beyond the range of facts reviewed here.

## Author Contributions

The author confirms being the sole contributor of this work and has approved it for publication.

## Conflict of Interest Statement

The author declares that the research was conducted in the absence of any commercial or financial relationships that could be construed as a potential conflict of interest.

## Footnotes

1 Declerck (1988, p. 79) also claims that the copula of a specificational pseudocleft cannot agree with the focus when this focus is an NPI, as in (i) (reproduced with Declerck’s judgments). But pseudoclefts with NPI-connectivity can be found for which at least some speakers allow for number agreement between the copula and the focus. The examples in (ii) (with (iic) taken from the internet) illustrate. (Here and throughout this paper, ‘^!^’ marks ‘unusual agreement.’) (i) what the book does not offer {is/*are} any solutions to the problems that are noted (ii) (a) what nobody has bought {is/^!^are} any cups and glasses (b) what isn’t available {is/^!^are} papers that say anything about clefts (c) what nobody has seen are any of the bonuses (including playmats, tokens, tins, counters) which were supposed to be shipped with the cards (http://boardgamegeek.com/thread/900269/stop-wulven-from-cheating-their-customers) In the analysis of NPI-connectivity in specificational pseudoclefts presented in Den Dikken et al. (2000), the examples in (i)–(ii) have as their post-copular constituent a full clause stripped down to the focus. If this is to involve constituent ellipsis (as effects of Merchant’s, 2001 P-stranding generalization in Dutch cases of this type suggest it must), we are dealing with a case of non-*wh* sluicing (IP ellipsis), with the focused constituent in the left periphery of the answer clause: (iii) [_TopP_ [_Question_ *what nobody bought*] [_Top_ Top = *be* [_Answer_ *any cups and glasses*_i_ [_IP_ *nobody bought t*_i_]]]] On this analysis, (i)–(ii) remind us of long-distance agreement in Tsez (Polinsky and Potsdam, 2001) and several other languages [incl. Innu-aimûn (Branigan and MacKenzie, 2002), Passamaquoddy (Bruening, 2001), and Itelmen (Bobaljik and Wurmbrand, 2005)]. The example in (ivb) illustrates long-distance agreement in Tsez: (iv) (a) eni-r [už-ā magalu mother-dat boy-erg bread.III.abs b-āc’-ru-łi] r-iyxo III-eat-ptc-nominal.IV IV -knows ‘the mother knows that they boy ate the bread’ (b) eni-r [už-ā magalu mother-dat boy-erg bread.III .abs b-āc’-ru-łi] b-iyxo III-eat-ptc-nominal.IV III -knows Polinsky and Potsdam (2001) argue that long-distance agreement in Tsez necessarily involves movement to an A′–position in the high left periphery of the subordinate clause — specifically, in the case of Tsez, SpecTopP, as in (v). The syntactic relationship between V and the lower topic in (v) is fully parallel to the relation between *be* and the lower focus in (iii). (v) V [_LowerClause_ *magalu*_i_ [_IP_ *užā t*_i_ *bāc’rułi*]]

2 Occurrences of number agreement in double-NP specificational copular sentences between the copula and the post-copular subject of predication are nonetheless attested. The examples in (i) (from Frances, 1986, p. 315) present two recorded cases of this type. (i) (a) the weather to watch *are* those rains (b) the cause of layoffs such as these *are* not the taxes

3 For sentences like *dat het echte probleem jij/jullie*___ ‘that the real problem you (SG/PL) BE’, my own intuitions reveal that ineffability arises precisely where the form of the copula is *explicitly* person-marked (present-tense 2SG *bent* vs 3SG *is*), while the result is acceptable with forms that are syncretic for person (*jij was* and *jullie zijn/waren*). Hartmann & Heycock’s (under review) experiments, revealing no person or syncretism effects here, may not have been sufficiently fine-grained to pick them up. These effects are perhaps even stronger in clefts (see also Ackema and Neeleman, 2018, who report intuitions matching mine): *dat het jij/jullie*___ *die S* ‘that it you (SG/PL) BE who S’ works with *was* (for *jij*) and *zijn/waren* (for *jullie*), but not with explicitly 2SG *bent*. Hereinafter, I will base myself on my own judgements for Dutch.

4 For the text account, it is important that ‘∅’ not be taken to be *pro* (which is plainly in possession of φ-features) but a silent noun (PERSON or THING, *à la* Kayne, 2005). See also Den Dikken and Griffiths (to appear) for relevant discussion. The English equivalents of (10)–(11) do not give rise to ineffability, thanks to the fact that English allows for *default* (3SG) inflection on the copula. Béjar and Kahnemuyipour (2017) argue that in Eastern Armenian and Persian sentences of the type *the problem is the children*, the copula has default inflection as well. In Dutch this is impossible.

5 Dillon et al. (2017, p. 90) point out that the Kimball and Aissen effect [at least in *wh*-questions, such as (13b)] ‘stands apart from other forms of attraction [such as (14)], either in strength or in kind.’ In “Why Person Is Different,” it will turn out that the syntax of the attraction configuration in (13) is also different in detail from that in (14).

6 Baker (2011) notes this for the Kimball and Aissen facts (15) (see also Dillon et al., 2017, whose test items consistently feature a form of *be* as the agreeing auxillary, *pace* Kayne, 2005, p. 264), and Nevins (2011) for ‘agreement attraction’ cases of the type in (16), both focusing on the first-person singular pronoun. I gave examples with second-person *you* as well to avoid interference coming from the case form of the pronoun — only *me*, not *I*, is possible in (4); and since *me* is not nominative, it will experience more difficulty in controlling agreement for independent reasons (see esp. Hartsuiker et al.’s, 2003 observation that the rate of agreement attraction is highest in cases in which the attractor is explicitly nominative or syncretic with the nominative). The second-person pronoun *you* shows case syncretism for nominative and accusative, hence should in principle be eligible for attracting agreement. The fact that it nonetheless fails to so do in (16b) is therefore interesting and significant. In this connection, note also the Hungarian examples in (i), where the possessive pronoun has the same form as the pronominal subject of a finite clause (‘nominative,’ or absence of morphological case-marking), yet person-agreement attraction remains sharply ungrammatical. (i) (a)az én identitásom titok {*volt*/**voltam*} (Hungarian) the I identity.1sg secret was(3sg)/was.1sg (b) a te identitásod titok {*volt*/**voltál*} the you identity.2sg secret was(3sg)/was.2sg ‘my/your identity was a secret’

7 Absence of any specification for person is often a viable option for ‘third person’ (Benveniste, 1966; Harley and Ritter, 2002*;* Nevins, 2007; Harbour, 2016; Ackema and Neeleman, 2018); but for English, third person is arguably D_[–PART]_. Relevant here is the discussion (in the section entitled “The Featural Specification of *Who* as Relative Operator”) of the English relative operator *who* as radically unspecified for person.

8 For my purposes here, it is immaterial whether N is inherently endowed with a categorial feature or has its category label determined by a *n*-head merged outside NP. In the latter case, it will be *n*, not N, that is endowed with the gender feature. I have very little to say in this paper about gender agreement (attraction). If gender agreement involves feature valuation in syntax, it should behave very much like number agreement: both ind and class are present on D. But I am not convinced that there is a gender probe on the clausal spine. I will proceed on the assumption that gender agreement involves concord (on which see “Relativization and Agreement”). For experiments and discussion (incl. a literature review) of gender agreement attraction effects (with some surprising results from Russian), I refer the reader to Slioussar and Malko (2016).

9 On why a DP cannot serve as the specifier of the constituent occupying the complement position of a higher D, see Den Dikken and Dékány (2018).

10 From Postal (1966), I retain the idea that the pronoun in *we/us/you linguists* occupies D — not through base-insertion (as Postal had it) but via raising. For a related proposal regarding the syntax of ‘pluringulars’ (as in *the committee are deliberating*), see Den Dikken (2001). See also Spanish ‘unagreement,’ briefly discussed in fn. 28, below.

11 A reviewer mentions that in Cheke Holo (an Oceanic language spoken on the Solomon Islands; see Bosma, 1981; Palmer, 2009), first- and second-person pronouns can co-occur with determiners (see, e.g., *ta-hati-a* ‘we_INCL_-pl-art’). A cursory inspection of the data suggests to me that this happens only when they are emphasized (with the emphasis particle *egu*, even in vocatives) or focused (with the particle *si*, as in *si iago ia* ‘foc you_SG_ art’), although appearing with *si* does not seem to require the presence of the article (*si go-tilo* ‘foc you-pl’). Whatever the determinants of the presence of the article with first-/second-person pronouns in Cheke Holo may turn out to be, it is noteworthy that articled personal pronouns do not seem to trigger person agreement on the finite verb in this language. This may follow from the proposal presented in this paper: when #P is encapsulated in a DP, the πP in Spec#P becomes very difficult (perhaps impossible) to access as an Agree-goal for the clausal π-head.

12 See esp. Preminger (2011) for a defence of this structure, aimed, like the present paper, at an understanding of SCOPA and the restrictions on person agreement. Hartmann & Heycock (under review) likewise have number and person project autonomously, but they follow Sigurðsson and Holmberg’s (2008) lead in placing person above number.

13 Here and in what follows, whenever I talk about ‘person,’ I am referring to first- or second-person. Recall that ‘third person,’ whenever it involves an explicit feature specification [–part], is marked on D, not on π; ‘third person pronouns’ are DPs, behaving in relevant respects like common noun phrases.

14 Preminger (2009; 2011, p. 920) discusses examples of long-distance agreement from ‘substandard Basque’ which he takes to instantiate person-feature valuation under downward Agree. I do not have the space here to engage in a discussion of these examples. I would seek to reanalyse them in terms of object shift into the matrix clause, with person valuation under the Spec–Head relation.

15 If the intervening feature had been valued antecedently, # would have had no trouble probing past it. But since (for reasons discussed in the previous paragraph) π cannot probe and value its unvalued feature by itself, this causes this feature to be a harmful intervener for the establishment of probe–goal relations by functional heads higher on the clausal spine. Taking π out of the way (by raising it up to #) is the only way around this intervention effect.

16 For a defense of the idea that probe–goal relations open up otherwise opaque domains, see Den Dikken (2018).

17 For Indo-European, it is not difficult to argue that finite C is specified for person in the same way that D is: the finite complementisers of Indo-European derive from nominal elements (demonstratives, *wh*-words). In the Loka̧a̧ examples in (17), there is no C-element to which we can attribute properties on independent grounds. But the fact that we are dealing with a gerund (well-known to be a hybrid of nominal and clausal properties) makes it plausible to assume that its C is specified for ‘third person.’ I will ignore the gender (noun-class) agreement found in (17). See fn. 8 for some remarks on gender agreement.

18 The familiar long-distance number/gender agreement cases of Tsez [from Polinsky and Potsdam, 2001; see (i)] involve a clause in complement (rather than subject) position. See Den Dikken (2018) for discussion of how this long-distance agreement comes about. (i) eni-r [už-ā magalu b-āc’-ru-łi] b-iyxo mother-dat boy-erg bread.III .abs III-eat-ptc-nominal.IV III -knows ‘the mother knows that they boy ate the bread’

19 The *there*-existential in (ib) makes the same point, given a predicate inversion approach to *there*-sentences (Hoekstra and Mulder, 1990). (ia) with *are* is identical with a naturally occurring sentence taken from the internet; but in (ib) *are* is entirely impossible. (i) (a)some people’s information {*is*/^!^*are*} stored in a database (b) there {*is*/**are*} some people’s information stored in a database

20 For the sake of uniformity, (37) gives a triple of non-restrictive relatives. Kimball and Aissen’s (1971) original examples involve restrictive relatives; but with first- and second-person heads, only non-restrictives are possible. As Baker (2011, p. 887, fn. 10) points out, number attraction to plural is possible in non-restrictive relatives as well.

21 Douglas (2015, p. 46, fn. 6) does in fact mention one the Kimball and Aissen facts in passing, but never draws these data into the analysis.

22 If one considers connectivity effects (for binding and idiomaticity) to furnish the only compelling argument for a raising approach to relativization, this conclusion, in the narrow version of the text formulation, is innocuous: relative clauses with a first- or second-person head cannot exhibit any such connectivity effects. But if one considers the distribution of articles with particular noun-phrase types to be a critical argument for the raising analysis (as Kayne, 1994 does), the text conclusion is more consequential: (ib) patterns with (ia) in this regard. (i) (a) the Paris *(that I knew as a boy) (b) the me *(that people don’t often see) Readers who, based on their general theoretical assumptions regarding the locality restrictions on agreement relations, have a different perspective regarding the possibility of person and number agreement between the structures in (47) and the finite verb of the relative clause will still come to the same *general* conclusion drawn here, viz., that the raising analysis is not capable of accounting for the person agreement facts in non-restrictive relative clauses with a first-person pronominal head. For me, it is (39) rather than (38) that stands out as the problem; but for those readers who find that (47) cannot control person agreement inside the relative clause, the problem is (38) rather than (39). Either way, the raising analysis is bound to run into trouble in the account of (38) vs. (39).

23 The Russian examples in this section were kindly provided by Irina Burukina (p.c.).

24 The facts of person agreement in German relative clause constructions are more complex than the English ones. Heck and Cuartero (2012) report a singular/plural split for finite verb agreement in relative clauses with a pronominal head: see (i). It is likely that the form of the relative pronoun plays a role in this, as Heck and Cuartero (2012) suggest. But the exact way in which the text analysis can mobilize the form of the German relative pronouns in an analysis of the agreement facts in (i) is something that I have not figured out at this time. (i) (a) *weil du es bist, der die ganze Arbeit machst (German) since you_SG_ it are.2sg relpron the whole work do.2SG (b) weil ihr es seid, die die ganze Arbeit macht since you_PL_ it are.2pl relpron the whole work do.2PL ‘since it’s you who do all the work’

25 One might ask whether concord between the head and the relative CP is possible at all when the I-domain is in a feature-valuing Agree relationship with a non-relativized subject. If C and I engage in an automatic feature-sharing relationship (‘feature inheritance,’ ‘head-head agreement’) and if feature-sharing between a head and its maximal projection is likewise automatic (as is standardly assumed: it is in fact a linchpin of the X-bar principle), it follows that when I is in an Agree relationship for its φ- and case-features with a non-relativized subject, it is impossible for the head and the relative CP to engage in concord for any features that do not match those of the subject of the relative clause. I will accept this conclusion, leaving a fuller investigation of the workings of concord for another occasion.

26 The unusual agreement pattern in (37c) is particularly well-known for cases of relativization, but Kimball and Aissen (1971) themselves also report such effects for root *wh*-questions: recall (13b). I will turn to this at the end of this section. Note in this connection as well that Hartsuiker et al. (2001) point out agreement attraction effects triggered by objects in Dutch SOV constructions.

27 General assumptions regarding the directionality of syntactic structure building (see Den Dikken, 2018 and references cited there) will make particular approaches to the way in which *who* in (37c) establishes its number agreement relation with the finite verb more plausible than others. But this is not the place to delve into these matters. All three approaches can, it seems to me, account for Kimball and Aissen’s (1971) observation that number agreement between the non-subject relative operator and the finite verb cannot happen when the subject of the relative clause is non-pronominal: (i). There are good grounds for believing that weak pronominal arguments must establish a Spec–Head relationship with the verb (as witness, for instance, the fact that object shift, which establishes a Spec–Head relation, is obligatory, in languages that have it, whenever the object is a weak pronoun). Forging an agreement relationship between *who* and the finite verb (whether it be via the Spec–Head relation or under Agree) would make it impossible for *he* to engage in a Spec–Head agreement relationship with the inflectional system of the relative clause. (i) these people, who he {is/*are} hoping will be the finalists, .. I would like to point out in this context that though Dillon et al. (2017, p. 81) did find ‘a non-significant numerical trend’ in this direction in object *wh*-questions (such as *which basketball players* {*is*/^!^*are*} *he planning to use this season?*), they ‘failed to observe any interaction of subject type [pronoun vs. lexical noun phrase; MdD] and the mismatch effect.’ In their conclusion (p. 90), however, they call attention to the fact that the approach to the attraction facts that they favor ‘predicts that highly marked controllers should outcompete less marked controllers, minimizing the amount of interference they contribute. Intuition suggests that this prediction is correct’ — based on examples of the type in (ii) [their (9), not explicitly tested in any of their experiments], involving a first-person singular subject. (ii) which flowers (am/*are) I planting in the garden today?

28 In their title and in various places throughout their paper, they refer to this as ‘object agreement.’ But as is clear from cases of the type in (54) (where the controller of finite verb agreement in the matrix clause is not an object of that clause but instead the subject of the subordinate finite clause), this term should not be taken too literally.

29 What Baker (2011) calls ‘two-and-a-half agreement’ (found in ditransitive constructions in which V agrees for all φ-features with the subject and the indirect object but cannot agree for person with the direct object) falls out from the text proposal: the direct object of a ditransitive, unlike the subject and the indirect object, is never in a Spec–Head relation with the verb. Baker’s (2011, sect. 3.1) analysis of partial agreement in Sakha is also directly compatible with my derivation of SCOPA. I will not talk here about the fact that a Spanish predicate adjective can reflect the number and gender features of its subject but never its person feature [Spanish *somos gorda(*mo)s* ‘we are fat’]. Baker derives this from SCOPA; for me this does not involve agreement but concord, which for reasons unknown to me can only be partial in this context. For the apparent person mismatch in Spanish ‘unagreement’ constructions (*los profesores somos inteligentes* ‘the professors are.1PL intelligent’), DP-internal predication between the common noun phrase and a silent pronoun (contributing ‘1PL’) is plausible. Mancini et al. (2014a) present three eye-tracking experiments and a grammaticality judgment task contrasting Spanish ‘unagreement’ cases and erroneous person agreement, showing that although ‘[s]imilarly to Person Mismatch, Unagreement elicited an early negative effect, suggesting rapid recognition of a subject–verb mismatch …, [a]t later stages, the effect of true person anomalies persisted, while the patterning of Unagreement with Standard Agreement clearly evidenced that the grammaticality of the apparent mismatch had been acknowledged’ (p. 143).

30 Mancini et al. (2017) is currently the most recent installment in a series of psycho- and neuro-linguistic studies conducted by Mancini et al. (2011; 2014a; 2014b, for earlier reports), all of which have found significant differences in behavior between number agreement and person agreement.

## References

[B1] Ackema P. Neeleman A. (2018). *Features of Person: From the Inventory of Persons to Their Morphological Realization.* Cambridge, MA: MIT Press.

[B2] Akmajian A. (1970). On deriving cleft sentences from pseudo-cleft sentences. *Linguist. Inq.* 1 149–168.

[B3] Baker M. (2008). *The Syntax of Agreement and Concord.* Cambridge: Cambridge University Press.

[B4] Baker M. (2011). When agreement is for number and gender but not person. *Nat. Lang. Linguist. Theory* 29 875–915. 10.1007/s11049-011-9147-z

[B5] Béjar S. Kahnemuyipour A. (2017). Non-canonical agreement in copular clauses. *J. Linguist.* 53 463–499. 10.1017/s002222671700010x

[B6] Benveniste E. (1966). *Problèmes de Linguistique Générale.* Paris: Gallimard.

[B7] Bianchi V. (1999). *Consequences of Antisymmetry: Headed Relative Clauses.* Berlin: Mouton de Gruyter.

[B8] Bobaljik J. Wurmbrand S. (2005). The domain of agreement. *Nat. Lang. Linguist. Theory* 23 809–865. 10.1007/s11049-004-3792-4

[B9] Bock K. Miller C. (1991). Broken agreement. *Cognit. Psychol.* 23 45–93. 10.1016/0010-0285(91)90003-7 2001615

[B10] Bosma D. (ed.) (1981). *Life in our Village: Short Stories From Solomon Islands.* Honiara: Translation Committee, Solomon Association.

[B11] Branigan P. H. MacKenzie M. (2002). Altruism, A’–movement, and object agreement in Innu-aimûn. *Linguist. Inq.* 33 385–407. 10.1162/002438902760168545

[B12] Bruening B. (2001). *Syntax at the Edge: Cross-Clausal Phenomena and the Syntax of Passamaquoddy*. Ph.D. thesis, MIT, Cambridge.

[B13] Declerck R. (1988). *Studies on Copular Sentences, Clefts and Pseudo-Clefts.* Leuven: Leuven University Press.

[B14] Den Dikken M. (1998). Review of Moro (1997). *Linguistische Berichte* 174 246–263.

[B15] Den Dikken M. (2001). ‘Pluringulars’, pronouns and quirky agreement. *Linguist. Rev.* 18 19–41.

[B16] Den Dikken M. (2006). *Relators and Linkers.* Cambridge, MA: MIT Press.

[B17] Den Dikken M. (2017). “Pseudoclefts and other specificational copular sentences,” in *The Companion to Syntax*, 2nd Edn, eds Everaert M. van Riemsdijk H. (Oxford: John Wiley & Sons, Inc.).

[B18] Den Dikken M. (2018). *Dependency and Directionality.* Cambridge: Cambridge University Press.

[B19] Den Dikken M. Dékány E. (2018). A restriction on recursion. *Syntax* 21 37–71. 10.1111/synt.12149

[B20] Den Dikken M. Dékány E. (2019). Adpositions and case: alternative realisation and concord. *Finno-Ugric Lang. Linguist*. 8.

[B21] Den Dikken M. Meinunger A. Wilder C. H. (2000). Pseudoclefts and ellipsis. *Stud. Linguist.* 54 41–89. 10.1111/1467-9582.00050

[B22] Den Dikken M. Griffiths J. (to appear). Ellipsis and Spec–Head agreement.

[B23] Dillon B. Levy J. Staub A. Clifton C. (2017). Which noun phrases is the verb supposed to agree with? Object agreement in American English. *Language* 93 65–96. 10.1353/lan.2017.0003

[B24] Dillon B. Mishler A. Slogget S. Phillips C. (2013). Contrasting intrusion profiles for agreement and anaphora: experimental and modeling evidence. *J. Mem. Lang.* 69 85–103. 10.1016/j.jml.2013.04.003

[B25] Douglas J. (2015). Agreement (and disagreement) among relatives. *Cam. Occas. Papers Linguist.* 7 33–60.

[B26] Frances W. N. (1986). Proximity concord in english. *J. English Linguist.* 19 309–318.

[B27] Franck J. Lassi G. Frauenfelder U. Rizzi L. (2006). Agreement and movement: a syntactic analysis of attraction. *Cognition* 101 173–216. 10.1016/j.cognition.2005.10.003 16360139

[B28] Guasti M. T. Rizzi L. (2002). “Agreement and tense as distinctive syntactic positions: Evidence from acquisition,” in *Functional Structure in DP and IP. The Cartography of Syntactic Structures*, Vol. 1 ed. Cinque G. (New York, NY: Oxford University Press), 167–194.

[B29] Harbour D. (2016). *Impossible Persons.* Cambridge, MA: MIT Press.

[B30] Harley H. Ritter E. (2002). Person and number in pronouns: a feature-geometric analysis. *Language* 78 482–526. 10.1353/lan.2002.0158

[B31] Hartsuiker R. J. Antón-Méndez I. van Zee M. (2001). Object attraction in subject-verb agreement construction. *J. Mem. Lang.* 45 546–572. 10.1006/jmla.2000.2787 16360139

[B32] Hartsuiker R. J. Schriefers H. Bock K. Kikstra G. M. (2003). Morphophonological influences on the construction of subject-verb agreement. *Mem. Cogn.* 31 1316–1326. 10.3758/BF03195814 15058692

[B33] Heck F. (2008). *On Pied-Piping: Wh-Movement and Beyond.* Berlin: De Gruyter Mouton.

[B34] Heck F. Cuartero J. (2012). “Long distance agreement in relative clauses,” in *Local Modelling of Non-Local Dependencies in Syntax*, eds Alexiadou A. Kiss T. Müller G. (Berlin: Mouton de Gruyter), 49–83.

[B35] Heycock C. (1992). *Layers of Predication. The Non-Lexical Syntax of Clauses*. Ph.D. thesis, University of Pennsylvania, New York, NY.

[B36] Heycock C. (2009). Agreement in specificational sentences in Faroese. *Nordlyd* 36 57–77.

[B37] Heycock C. (2012). Specification, equation, and agreement in copular sentences. *Can. J. Linguist.* 57 209–240. 10.1353/cjl.2012.0033

[B38] Hoekstra T. Mulder R. (1990). Unergatives as copular verbs: locational and existential predication. *Linguist. Rev.* 7 1–79.

[B39] Kayne R. (2005). *Movement and Silence.* New York, NY: Oxford University Press.

[B40] Kayne R. S. (1994). *The Antisymmetry of Syntax.* Cambridge, MA: MIT Press.

[B41] Kayne R. S. (1998). *Class lectures, NYU, fall 1998.*

[B42] Kayne R. S. (2000). “Notes on English agreement,” in *Parameters and Universals*, ed. Kayne R. S. (Oxford: Oxford University Press), 187–205.

[B43] Kimball J. Aissen J. (1971). I think, you think, he think. *Linguist. Inq.* 2 241–246.

[B44] Mancini S. Molinaro N. Rizzi L. Carreiras M. (2011). A person is not a number: discourse involvement in subject–verb agreement computation. *Brain Res.* 1410 64–76. 10.1016/j.brainres.2011.06.055 21798519

[B45] Mancini S. Molinaro N. Davidson D. Avilés A. Carreiras M. (2014a). Person and the syntax–discourse interface: an eye-tracking study of agreement. *J. Mem. Lang.* 76 141–157. 10.1016/j.jml.2014.06.010

[B46] Mancini S. Postiglione F. Laudanna A. Rizzi L. (2014b). On the person-number distinction: subject-verb agreement processing in Italian. *Lingua* 146 28–38. 10.1016/j.lingua.2014.04.014

[B47] Mancini S. Quiñones I. Molinari N. Hernandez-Cabrera J. Carreiras M. (2017). Disentangling meaning in the brain: left temporal involvement in agreement processing. *Cortex* 86 140–155. 10.1016/j.cortex.2016.11.008 27984788

[B48] Merchant J. (2001). *The Syntax of Silence: Sluicing, Islands, and the Theory of Ellipsis.* Oxford: Oxford University Press.

[B49] Morgan J. L. (1972). “Verb agreement as a rule of English,” in *Papers From the Eighth Regional Meeting of the Chicago Linguistic Society*, eds Peranteau P. M. Levi J. N. Phares G. C. (Chicago: CLS), 278–286.

[B50] Moro A. (1997). *The Raising of Predicates: Predicative Noun Phrases and the Theory of Clause Structure.* Cambridge: Cambridge University Press.

[B51] Nevins A. (2007). The representation of person and its consequences for person–case effects. *Nat. Lang. Linguist. Theory* 25 273–313. 10.1007/s11049-006-9017-2

[B52] Nevins A. (2011). Multiple agree with clitics: person complementarity vs. omnivorous number. *Nat. Lang. Linguist. Theory* 29 939–971. 10.1007/s11049-011-9150-4

[B53] Palmer B. (2009). Clause order and information structure in Cheke Holo. *Ocean. Linguist.* 48 213–249. 10.1353/ol.0.0038

[B54] Polinsky M. Potsdam E. (2001). Long-distance agreement and topic in Tsez. *Nat. Lang. Linguist. Theory* 19 583–646.

[B55] Postal P. (1966). “On so-called ‘pronouns’ in English,” in *Report on the Seventeenth Annual Round Table Meeting on Linguistics and Languages Studies*, ed. Dinneen F. (Washington, DC: Georgetown University Press), 177–206.

[B56] Preminger O. (2009). Breaking agreements: distinguishing agreement and clitic-doubling by their failures. *Linguist. Inq.* 40 619–666. 10.1162/ling.2009.40.4.619

[B57] Preminger O. (2011). Asymmetries between person and number in syntax: a commentary on baker’s SCOPA. *Nat. Lang. Linguist. Theory* 29 917–937. 10.1007/s11049-011-9155-z

[B58] Raposo E. (1987). Case theory and Infl-to-comp: the inflected infinitive in European Portuguese. *Linguist. Inq.* 18 85–109.

[B59] Ross J. R. (1970). “On declarative sentences,” in *Readings in English Transformational Grammar*, eds Jacobs R. A. Rosenbaum P. S. (Waltham, MA: Ginn and Company), 222–277.

[B60] Schütze C. (2001). On the nature of default case. *Syntax* 4 205–238. 10.1111/1467-9612.00044

[B61] Shlonsky U. (2004). The form of semitic noun phrases. *Lingua* 114 1465–1526. 10.1016/j.lingua.2003.09.019

[B62] Sigurðsson H. Á Holmberg A. (2008). “Icelandic dative intervention: Person and Number are separate probes,” in *Agreement Restrictions*, eds D’Alessandro R. Fischer S. Hrafnbjargarson G. H. (Berlin: Mouton de Gruyter), 251–279.

[B63] Slioussar N. Malko A. (2016). Gender agreement attraction in Russian: production and comprehension evidence. *Front. Psychol.* 7:1651. 10.3389/fpsyg.2016.01651 27867365 PMC5095607

[B64] Uriagereka J. (1999). “Multiple spell-out,” in *Working Minimalism*, eds Epstein S. D. Hornstein N. (Cambridge, MA: MIT Press), 251–282.

[B65] Wechsler S. (2011). Mixed agreement, the person feature, and the index/concord distinction. *Nat. Lang. Linguist. Theory* 29 999–1031. 10.1007/s11049-011-9149-x

